# Structure and Assembly of a Trans-Periplasmic Channel for Type IV Pili in *Neisseria meningitidis*

**DOI:** 10.1371/journal.ppat.1002923

**Published:** 2012-09-13

**Authors:** Jamie-Lee Berry, Marie M. Phelan, Richard F. Collins, Tomas Adomavicius, Tone Tønjum, Stefan A. Frye, Louise Bird, Ray Owens, Robert C. Ford, Lu-Yun Lian, Jeremy P. Derrick

**Affiliations:** 1 Faculty of Life Sciences, Michael Smith Building, University of Manchester, Manchester, United Kingdom; 2 Institute of Integrative Biology, University of Liverpool, Liverpool, United Kingdom; 3 Centre for Molecular Biology and Neuroscience, University of Oslo, Oslo, Norway; 4 Oxford Protein Production Facility, Research Complex at Harwell, Harwell, Oxford, United Kingdom; Faculté de Médecine Paris Descartes, site Necker, France

## Abstract

Type IV pili are polymeric fibers which protrude from the cell surface and play a critical role in adhesion and invasion by pathogenic bacteria. The secretion of pili across the periplasm and outer membrane is mediated by a specialized secretin protein, PilQ, but the way in which this large channel is formed is unknown. Using NMR, we derived the structures of the periplasmic domains from *N. meningitidis* PilQ: the N-terminus is shown to consist of two β-domains, which are unique to the type IV pilus-dependent secretins. The structure of the second β-domain revealed an eight-stranded β-sandwich structure which is a novel variant of the HSP20-like fold. The central part of PilQ consists of two α/β fold domains: the structure of the first of these is similar to domains from other secretins, but with an additional α-helix which links it to the second α/β domain. We also determined the structure of the entire PilQ dodecamer by cryoelectron microscopy: it forms a cage-like structure, enclosing a cavity which is approximately 55 Å in internal diameter at its largest extent. Specific regions were identified in the density map which corresponded to the individual PilQ domains: this allowed us to dock them into the cryoelectron microscopy density map, and hence reconstruct the entire PilQ assembly which spans the periplasm. We also show that the C-terminal domain from the lipoprotein PilP, which is essential for pilus assembly, binds specifically to the first α/β domain in PilQ and use NMR chemical shift mapping to generate a model for the PilP:PilQ complex. We conclude that passage of the pilus fiber requires disassembly of both the membrane-spanning and the β-domain regions in PilQ, and that PilP plays an important role in stabilising the PilQ assembly during secretion, through its anchorage in the inner membrane.

## Author Summary

Many bacteria which cause infectious disease in humans use large fibers, called pili, to attach to the surfaces of the cells of the host. Pili are also involved in a particular type of movement of bacteria, termed twitching motility, and the uptake of DNA into the bacterial cell. They are made up of thousands of copies of a specific pilin protein. The process of assembly of pili is complicated: it requires the cooperative action of a group of proteins which span both the inner and outer membranes in bacteria. Here we have determined the structure of part of the machinery which forms a channel between both membranes. One of the proteins, PilQ, is organized in a segmental way, being divided into separate domains which are jointed, hence allowing them to move relative to each other. We infer that this movement is critical to the functioning of the channel, which must open up to allow passage of the pilus fiber. We suggest that the function of the other protein we have studied, PilP, is to maintain the PilQ assembly during pilus secretion.

## Introduction

Type IV pili are long (1–5 µm), mechanically strong polymers which extend from the surfaces of many Gram-negative bacteria, including *Neisseria meningitidis*, *Pseudomonas aeruginosa* and *Vibrio cholerae* [1], [2]. They are known to mediate a variety of functions, including attachment to host cell surface receptors during infection [3], natural DNA competence [4] and a phenomenon termed twitching motility, a flagellum-independent process which enables some bacteria to move rapidly (1 µm/s^−1^) across surfaces [5]. The pilus fiber consists principally of subunits of pilin (PilE in *N. meningitidis*), a small protein which adopts an α/β fold and assembles into a helical structure which confers mechanical strength on the assembly [6], [7], [8]. Twitching motility is associated with a notable feature of type IV pili: an ability to retract rapidly at a rate of approximately 1,000 pilin subunits per second, generating a powerful mechanical force which has been measured at up to 100 pN per fiber [9], [10].

The secretins are a large and diverse family of integral outer membrane (OM) proteins which comprise key components of the type II and type III secretion systems, as well as the biogenesis systems for type IV pili and filamentous bacteriophage [11]. Three-dimensional reconstructions of secretin structure by electron microscopy have revealed that they adopt multimeric structures, characterized by the formation of large chambers which lie within the periplasm. Our previous work on PilQ from *Neisseria meningitidis* showed a dodecameric structure, with a chamber sealed at both ends [12]. Studies on the type II secretion system (T2SS) secretins PulD [13] and, more recently VcGspD which is responsible for the secretion of *V. cholerae* toxin, revealed a cylindrical-shaped structure with 12-fold symmetry enclosing a large chamber which is open at the periplasmic end but closed at the OM [14]. The structure of a type III secretion system (T3SS) secretin can also be extracted from the 10 Å resolution cryoelectron microscopy density map of the *Salmonella* needle complex: this shows the secretin in an open state, with the needle passing through both ends of the chamber [15].

**Figure 1:**
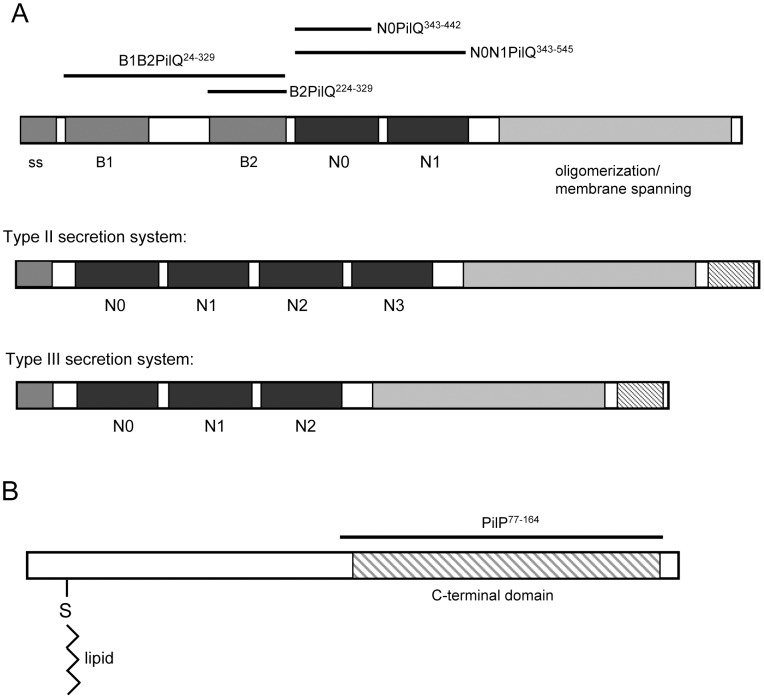
Schematic illustration of secretin and PilP domain structures. A) Secretin domain organisation: a type IV pilus-dependent secretin (PilQ; top) is shown compared to type II and type III secretion system secretins (middle and bottom). The proposed domain names are based on secondary structure predictions and homology sequence alignments. B1 and B2 represent structural regions predicted to be rich in β-structure; N0, N1, N2 and N3 represent the consecutively arranged α/β domains described previously for the GspD [20] and EscC [21] secretins. The C-terminal domain is responsible for secretin oligomerization and is embedded in the outer membrane. The ss shaded area is the signal peptide sequence. The constructs discussed in this paper are indicated above, including residue numbering based on the full length protein. B) PilP domain organisation: the lipid moiety is covalently linked to a conserved cysteine at the N-terminus, and is separated from the globular C-terminal domain by a region which is predicted is to be in an unstructured state.

Figure 1A shows a schematic illustration of the domain structure of *N. meningitidis* PilQ and two prototypical T2SS and T3SS secretins. All share a well conserved C-terminal region which spans the membrane and is responsible for oligomerization [13], [16], [17], [18], [19]. The central and N-terminal regions are more diverse; crystal structures of the N0, N1 and N2 domains from the T2SS and T3SS secretins have been reported, GspD [20] and EscC [21]. The structure of each domain is well conserved, and is based on a core fold of two α-helices packed against a three-stranded β-sheet. Docking of a model based on the N0/N1/N2 GspD crystal structure into the VcGspD cryoelectron microscopy electron density map established that these domains extend into the periplasm and form the sides of the secretin chamber [14].

A number of proteins are known to interact with secretins, either for the purposes of assembly, OM insertion or mediation of function once the mature protein has been formed. Pilotin proteins are responsible for membrane targeting of secretins: the interaction sites of some have been mapped to the extreme secretin C-terminus, and their recognition of T2SS and T3SS secretins has recently been revealed at the structural level [22], [23]. At least two proteins, PilW and Omp85, are known to promote assembly of the PilQ oligomer [24], [25]. Other proteins seem to play a more direct structural role: PilP is a lipoprotein which binds to PilQ and is essential for type IV pilus (TFP) formation [26], [27], [28]. It has an N-terminal lipid attachment site, followed by an unstructured N-terminal region and a C-terminal globular domain which adopts a lipochalin-like β-structure [29], [30] (Figure 1B). It has been shown to be located in the inner membrane [27]. Recent evidence has established that the fold of the PilP C-terminal domain is similar to that adopted by the HR domain from the T2SS GspC protein, which is known to bind to its cognate secretin, GspD [31]. A crystal structure of a complex between the GspC HR domain and the N0/N1 domains from GspD revealed a binding site formed by the edge-on association of β-strands from GspC and the GspD N0 domain [31]. PilP is among a group of ‘core’ proteins which are essential for assembly of TFP in *Neisseria meningitidis* [26]. Recent evidence from studies in *Pseudomonas* has shown that PilP also binds to PilO and PilN, two integral inner membrane proteins which are essential for pilus formation [30]. PilP thus forms a link between the OM, through its interaction with PilQ, and the inner membrane components of the type pilus biogenesis system. Unlike some other bacterial secretion systems, however, there is currently little structural information on the way in which the TFP biogenesis proteins assemble.

Here we report the structural determination of the PilQ periplasmic domains by using a combination of NMR and homology modelling. The original reconstruction of the PilQ oligomer which we reported was generated using cryonegative stain [12]; whilst this served to define the overall dimensions and structure of the complex, it cannot reliably be used for automated docking of constituent domains into the density map. We therefore also report a new 3D reconstruction of the PilQ oligomer, generated by single particle averaging from cryoelectron microscopy data of unstained specimens, and use this to dock the domain structures and generate the dodecameric assembly. Finally, we use a combination of NMR chemical shift perturbations and modelling to generate the complex formed between the first α/β domain in PilQ and the C-terminal domain of PilP. We propose that the segmental organization of the domain structure within PilQ is intrinsic to its ability to open up and form a channel to allow entry of the pilus fiber into the chamber, and its subsequent passage across the periplasm and OM.

## Results

### NMR structure of the PilQ β-domains

**Figure 2:**
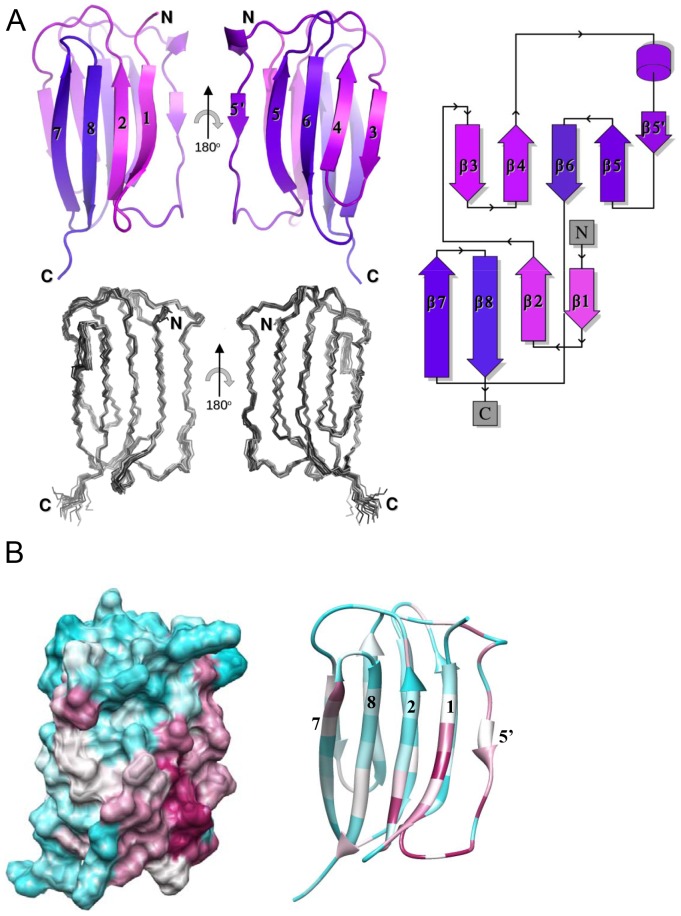
Structure of the *N. meningitidis* PilQ B2 domain. A) Two views of a ribbon plot (above) and structural ensemble (below) of the B2 domain (B2PilQ^224–329^). The ribbon plot and fold topology diagram (right), generated using Topdraw [62], are shown with a monochromatic gradient from the N- to C-terminus. B) Surface and ribbon plots of the β-domain, generated using CHIMERA [69], showing sequence conservation determined using CONSURF [35]. High sequence conservation is shown in purple, medium in white and low in light blue.

Bioinformatic studies suggested that the N-terminal regions of TFP-dependent secretins generally contained one or two putative domains, predicted to be rich in β-sheet and characteristically different from the α/β domains observed in T2SS and T3SS secretins [32]. We therefore adopted a cloning and expression strategy which over-produced these β-domains from TFP-dependent secretins originating from a number of different Gram-negative bacteria, including *N. meningitidis*, *P. aeruginosa*, *Aeromonas hydrophila, Xanthomonas campestris and Xylella fastidiosa*. We generally found the B2 domain more amenable to over-production and purification than B1 (Figure 1A), and obtained good quality NMR spectra from a construct spanning residues 224 to 329 in *N. meningitidis* PilQ (B2PilQ^224–329^; Figure 1A). NMR spectra of the ^13^C/^15^N uniformly labelled sample exhibited characteristic shifts of a well-folded predominantly β-strand structure, confirmed by ^1^H, ^13^C and ^15^N assignment of native sequence (92.3% complete). The solution structure of the second β-domain revealed an eight-stranded β-sandwich structure which is a novel variant of the HSP20-like fold (Figure 2A). The most similar fold identified within the SCOP database [33] is the CS domain from the human Sgt1 kinetochore complex [34]. The β-domain fold is larger, however, and includes two additional β-strands, such that β5 is paired with β6, rather than β4, as is the case with the CS domain (Figure S1).

A comparison of the sequences of the second β-domains from PilQ in different Gram-negative bacteria revealed a high degree of conservation within the region between β4 and β5, including the short β5′ strand (Figure S2). This observation was highlighted by application of the program CONSURF [35], which maps sequence conservation on to protein structure; in this case sequences from 63 different TFP-dependent secretins are mapped on to the surface of the B2 domain (Figure 2B). Strikingly, the most highly conserved residues map to a single patch on the domain surface, incorporating Lys232 from β1 with Asp281 and Phe282 from the β4/β5 loop. The implication is that this patch forms a binding site, possibly to another unidentified TFP biogenesis protein.

In contrast to the B2 domain, attempts to over-produce the B1 domains from several sources generally met with limited success: protein products were either produced in low yield and/or exhibited poor stability. The best progress was made with the B1 domain from *Aeromonas hydrophila:* assignment of the NMR spectra and use of chemical shift indices show that the *A. hydrophila* B1 domain consists of nine β-strands (Figure S3). The poor stability of this single domain precluded the collection of the high quality NOEs required for structural determination. Nevertheless, the similarities in secondary structure between the B1 and B2 domains determined by the NMR chemical shift indices suggest that they share a common origin, as seems to be the case with the repeated N0/N1/N2 domains within the N-terminal sections of the T2SS and T3SS secretins [11]. Most TFP-dependent secretins contain two β-domains, although the first β-domain is missing from some (eg *Xylella fastidiosa)*. It is noteworthy that residues which are highly conserved in the B2 domain (Figure 2B) are not found to be so in the B1 domain and *vice versa*. In addition, an interesting variation in neisserial PilQ is the presence of low complexity repeat sequences, termed small basic repeats (SBRs), which lie between the B1 and B2 domains and have been shown to influence the efficiency of TFP formation [36]. The presence of such polymorphic repeat elements is unprecedented within the secretin family. As we show below, electron density within the cryoelectron microscopy map for the whole PilQ oligomer cannot accommodate 12 copies of the B1 domain if it folds into a compact, globular structure similar to the B2 domain, so it may be the case that the B1 domains adopt a partially unfolded state in the assembled oligomer.

### NMR structure of the N0/N1-domains

**Figure 3:**
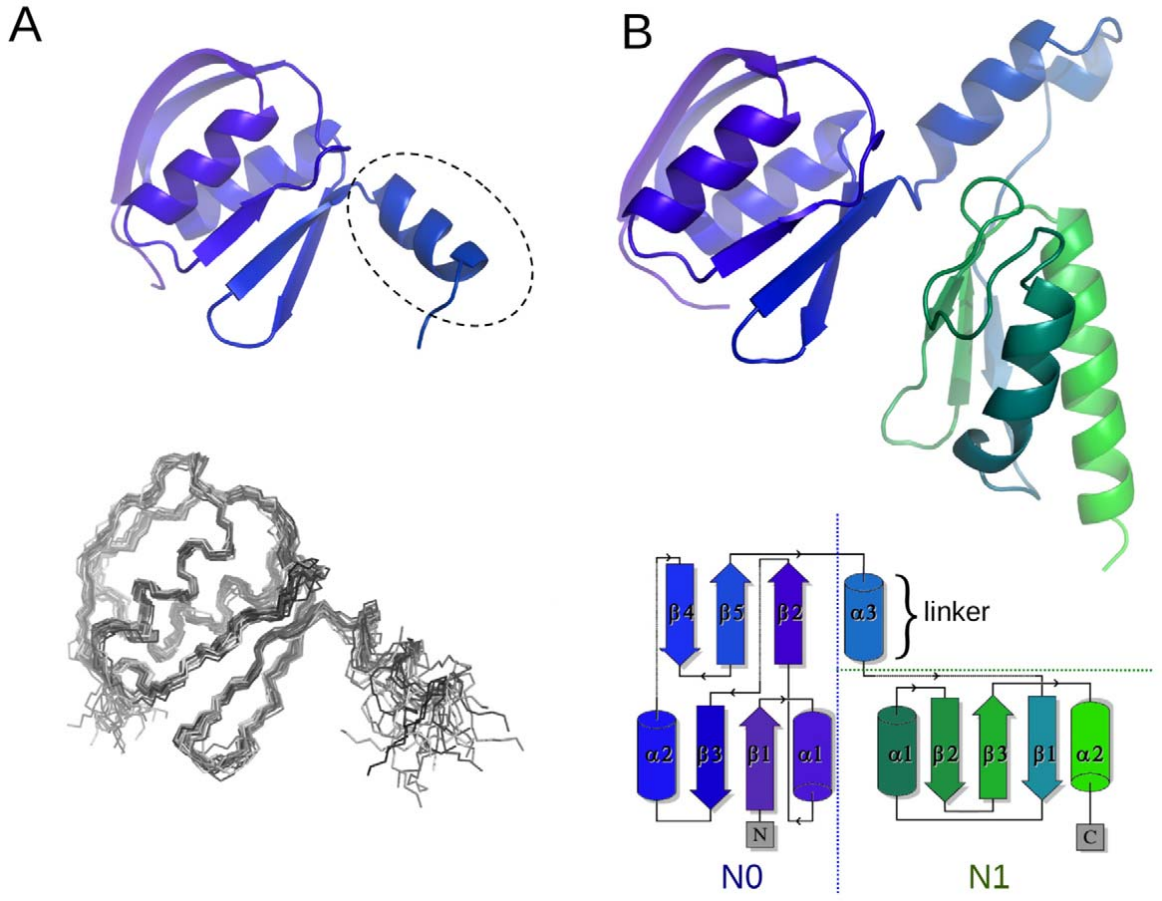
Structure of the *N. meningitidis* PilQ N0/N1 domains. A) Structure of the N0 domain (NmPilQ^343–442^), ribbon plot (above and structural ensemble (below); for clarity flexible residues 431–442 are omitted. The dashed circle indicates the proposed linker region. B) A composite model for the N0/N1 double domain structure (N0N1PilQ^343–545^), is shown with a indigo-green gradient from N- to C-terminus. The model is based on the NMR-derived structure for the first domain, and a CS-ROSETTA [65] model for the second domain and linker, with the relative orientation of the domains selected from optimal fit to the cryoelectron microscopy density map. The topology of the N0/N1 domains is outlined below, generated using TOPDRAW [62].

Secondary structure predictions and sequence alignments suggested the existence of two domains which are likely to adopt a variant of the α/β-type fold identified in other secretins [20], [21]. In a similar approach to that employed for the β-domains, single and multiple domain fragments from different bacteria were over-produced, purified and analysed by NMR. A two domain fragment from *N. meningitidis*, N0N1PilQ^343–545^ (Figure 1A), exhibited well dispersed NMR spectra: it was subsequently assigned and its secondary structure determined (Figure S4). Both the N0 and N1 domains are folded, but N1 contains a long random coil extension of over 35 amino acids at its C-terminal end. The very intense peaks from this region obscured many of the peaks from the folded domain of N1 and precluded extraction of the high quality NOEs required for a complete structure determination of the N0/N1 tandem domains. Using the Chemical Shift Index (CSI) information as a marker for the domain boundaries, a smaller fragment was produced which encompassed only the first domain (N0PilQ^343–442^) and its NMR structure determined by conventional methods using NOE restraints. The high quality structure adopts a fold similar to the N0 domains identified from GspD and EscC [20], [21] (Figure 3A; Table 1). Comparison of the spectra from the single and double domain protein samples verified that the chemical shifts from common residues in the first domain are very similar in both samples (not shown). A striking and novel feature of the domain structure is the presence of an α-helix at the C-terminus of this domain (circled in Figure 3A): from sequence alignments, this appears to be a general feature of the TFP-dependent secretins and is absent from other secretin types. The structure of the N1 domain was constructed using the CSI data, CS-ROSETTA and homology modeling, based on the crystal structure of the same domain from EscC [21] (Figure 3B).

**Table 1:** NMR structure calculation statistics.

*NMR constraints*	*B2PilQ^224–329^*	*N0PilQ^343–442^*
Total number of distance constraints		
Short range (|i-j|< = 1)	1190	807
Medium range (1<|i-j|<5)	274	207
Long range (|i-j|>5)	1144	296
***Structure statistics (20 structures)***		
Violation statistics		
Average number of NOE violations >0.3 Å	0	0
NOE violations >0.3 Å	0	0
Maximum NOE violation	0.29	0.29
***Ramachandran statistics (%)***		
Residues in most favoured regions	79.57	76.48
Residues in additional allowed regions	18.47	19.75
Residues in generously allowed regions	1.64	3.09
Residues in disallowed regions	0.36	0.72
***RMS deviations from the mean structure (Å)***		
Protein backbone*	0.33	0.64
Protein heavy atoms*	1.02	1.40

* RMSD based on residues 226–326 of *B2PilQ^224–329^* and 345–415 of *N0PilQ^343–442^*.

Analysis of the ^15^N-^1^H residual dipolar couplings (RDCs) indicated that the N0 and N1 domains have no fixed orientation relative to each other in solution: it was therefore not possible to obtain a common orientation in the alignment tensor frame for the N0 and N1 domains from the RDC measurements. However, the rotation correlation times, calculated from the ^15^N T_1_ and T_2_ values obtained separately for the single N0 domain (τ_c_∼9.6 ns) and the N0/N1 double domain (τ_c_∼14 ns), suggest that the N0 and N1 domains do not tumble completely independently. It is likely that the helical part of the linker between the two domains reduces the flexibility in this region. We therefore generated 100 structures of the N0/N1 double domain using CS-ROSETTA [37], with varying inter-domain orientations. The relevant section of the cryoelectron microscopy density map was then used to identify the cluster of structures which gave the best fit, as well as satisfying other constraints (see below). Interestingly, the relative orientation of the PilQ N0 and N1 domains bears a closer similarity to that observed in the T3SS secretin EscC [21], rather than the T2SS secretin GspD [20]. Clearly, crystal packing constraints and other factors can also influence relative domain orientations. Nevertheless, our observations do lend weight to the idea that the flexibility of the N0/N1 secretin domains could be an integral part of their function.

### Binding of the PilP C-terminal domain (PilP^77–164^) to the PilQ N0 domain

**Figure 4:**
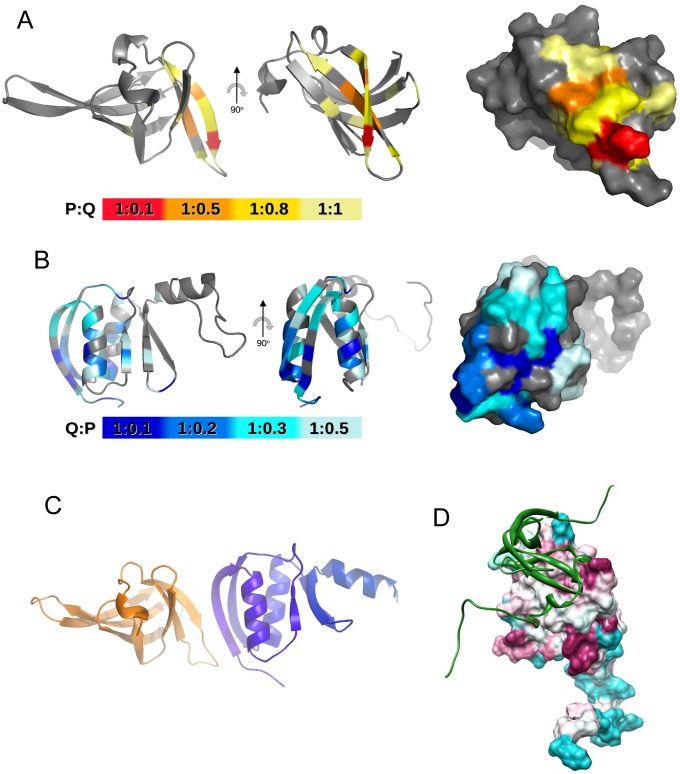
Structural model for the PilP C-domain bound to the PilQ N0 domain. A) Peak attenuation mapped on to the PilP C-domain (PDB accession 2IVW) following titration with N0N1PilQ^343–545^. Ratios of PilP:PilQ were colored as follows: 1∶0.1, red; 1∶0.5, orange; 1∶0.8, yellow; 1∶1, pale-yellow. Left, ribbon plot with β1 stand marked; right, surface plot. B) Peak attenuation mapped on to N0PilQ^343–442^. Ratios of PilQ:PilP were colored as follows: 1∶0.1, dark-blue; 1∶0.2, blue; 1∶0.3, cyan; 1∶0.5, pale-blue. C) Model of the PilP^77–164^: N0PilQ^343–442^ complex generated from CNS1.2 [56], with PilP^77–164^ in gold and N0PilQ^343–442^ in blue. Flexible residues at the N- and C-termini have been removed for clarity. D) Surface plot of the N0PilQ^343–442^ domain generated using CONSURF [35] and CHIMERA [69], with the same color scheme as used in Figure 2B. A ribbon plot of the PilP C-domain structure [29] is shown in green. The same sequence set was used for CONSURF as employed in Figure 2 B).

PilP^77–164^ is a recombinant fragment which corresponds to the C-terminal domain of the PilP lipoprotein (Figure 1B). Titration of unlabelled N0N1PilQ^343–545^ into ^15^N-labelled PilP^77–164^ identified, from the chemical shift changes [29], [38], a patch of residues on the PilP domain surface involved in binding. These were concentrated mainly into an area around the β1–β2 hairpin in the PilP^77–164^ structure (Figure 4A). The reverse experiment, where unlabelled PilP^77–164^ was titrated into ^15^N-labelled N0N1PilQ^343–545^, demonstrated that it is the N0 domain, rather than the N1 domain, which is involved in recognition of PilP^77–164^ (Figure S5). The experiment was repeated using the single N0 domain, N0PilQ^343–442^, and similar results were obtained. The largest chemical shift attenuations mapped to one side of the structure, concentrated around the first α-helix and β-strand in the fold of PilQ (Figure 4B). Similar experiments titrating PilP^77–164^ into the B1B2PilQ^24–329^ and B2PilQ^224–329^ domains did not show any evidence of binding (not shown).

The identified residues involved in binding on the surface of each protein were used as input into the restraint-driven docking programme HADDOCK [39], [40] to generate a structural model for the PilP^77–164^:N0N1PilQ^343–545^ complex. The largest HADDOCK-generated cluster bore marked similarities to the GspC-GspD complex [31]. However, upon further analysis of the HADDOCK-generated structures, a side chain was found to be artificially fixed in position by the rigid body docking procedure, interfering with the protein-protein interface. To allow for greater residue flexibility, the NMR restraints from N0PilQ^343–442^ and PilP^77–164^ (PDB 2IVW), together with five intermolecular edge-on backbone hydrogen bond restraints (derived from the favored HADDOCK structure and related GspC-GspD complex), were input into CNS1.2 [41] to generate the final model for the complex (Figure 4C). The binding site is centred around an edge-on interaction between the first two β-strands in each domain. Residue conservation was mapped on to the N0N1PilQ^343–545^ structure using CONSURF [35], in a similar manner to its implementation for the B2 domain (above), and provided evidence that the proposed binding site for PilP is moderately or well conserved within type IV pilus-dependent secretins (Figure 4D). We conclude that the C-terminal domain of PilP (Figure 1B) recognises the N0 domain from *N. meningitidis* PilQ in a similar manner to that for GspC and its cognate GspD secretin. This is therefore a further example of the congruence between the type II secretion and type IV pilus biogenesis systems.

### Cryoelectron microscopy structure of the PilQ dodecamer

**Figure 5:**
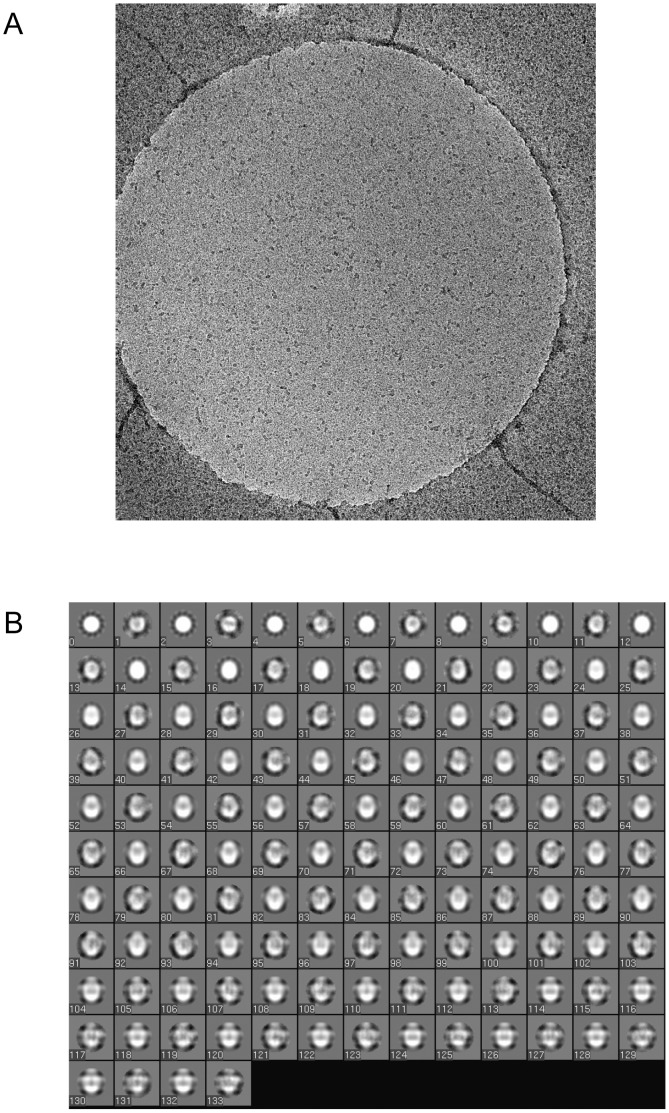
Determination of PilQ oligomer structure by single particle averaging: Raw data image and class averages. A. Frozen-hydrated specimen of PilQ complexes resuspended in a dodecyl maltoside-containing buffer at 1 mg/ml. The particles in the 1300 nm diameter hole in the carbon support film were selected interactively for image processing and single particle alignment, and then a preliminary model was generated by selecting projections with bilateral symmetry as well as 12-fold rotational symmetry. This model was then used as an alignment reference to which all particles were aligned, after which a refined model was generated. After several rounds of iterative refinement, no further improvement in the model was detected (as judged by Fourier Shell Correlation between the Nth and (N-1)th iteration). B. Comparison between different orientations of the final model (even numbered projections) and averages of all the particles best corresponding to those projections (subsequent odd numbered projection averages). Deviations between the odd and subsequent even numbered projections reflect the errors in the processing procedure, especially with respect to classification and rotational and translational alignment, and are a useful separate measure of the resolution and reliability of the structural data.

**Figure 6:**
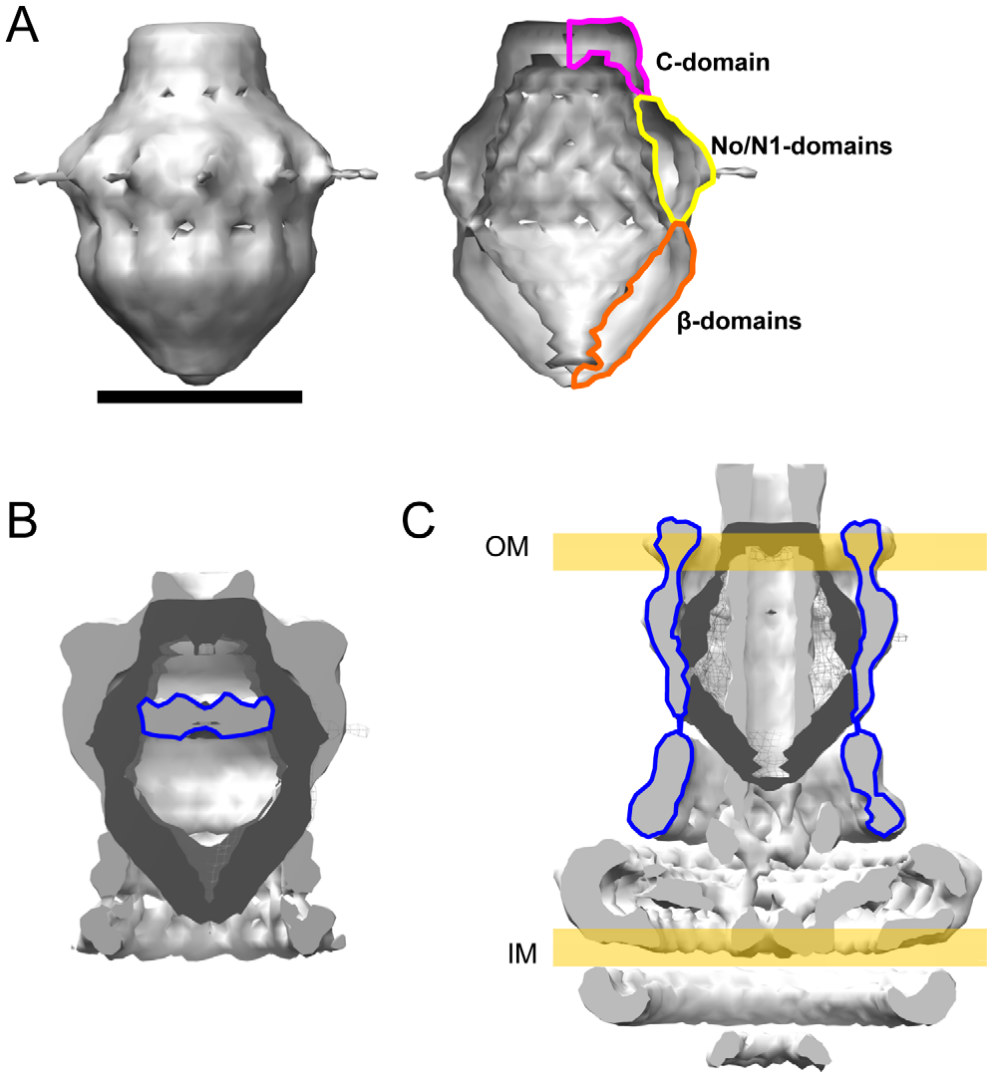
Cryoelectron microscopy structure of the PilQ dodecamer and comparison with *Salmonella* type III secretion system needle complex. A) Left panel: surface-contoured map; scale bar = 100 Å; right panel, as left, but with the front half of the volume removed to reveal major domain boundaries. B) Superposition of the PilQ density map (dark gray) onto *Vibrio cholerae* GspD (light gray; EMD-1763). The periplasmic gate structure is outlined in blue. C) Superposition of the PilQ density map (dark gray) onto *Salmonella* T3SS needle complex map (light gray; EMD-1875). The approximate locations of the outer membrane (OM) and inner membrane (IM) are shown and the density attributed to the InvG secretin is highlighted in blue.

We have previously reported on the structure of the intact *N. meningitidis* PilQ oligomer, using negative stain-based methods [12], [42]. This work established that PilQ forms a dodecamer, in common with the T2SS secretins [13], [14]. In order to generate a structure which would allow docking of the domain structures presented above, we determined a 3D reconstruction of the complete PilQ dodecamer by cryoelectron microscopy. PilQ particles were well dispersed and clearly identifiable (Figure 5A). Single particle selection of 25,303 particles generated a good range of top, side and intermediate views (Figure 5B). The final structure, measuring 155 Å in height and 110 Å at its widest external extent, forms a shell around a large internal chamber (Figure 6A). The chamber is sealed at both ends, and a cut-away view shows evidence for distinct and separate structures within the density map (marked on the right hand side of Figure 6A). From our previous work [12], [42], [43], and comparisons with the structures of other secretins, we ascribe the flattened disc of density at the top of the structure to the membrane-spanning C-terminal domain, which is highly conserved within the secretin family. Our work above has established that PilQ, in common with the other secretins, adopts a ‘string of beads’ type domain organisation. Combining this evidence, we deduce that the structure lining the walls of the chamber, outlined in yellow in Figure 6A, can be reasonably ascribed to the N0/N1 domains. The N-terminal region, encompassing the β-domains would, therefore, form the part of the oligomer which closes the chamber at the bottom (outlined in orange in Figure 6A).

Alignment of the PilQ density map with the T2SS secretin VcGspD [14] shows some key structural differences between the two. PilQ is more compact and, critically, closed at the base, where VcGspD has a flared, open gateway to the secretin chamber (Figure 6B). We attribute this difference to the presence of the B1 and B2 domains in PilQ, which are absent from VcGspD (Figure 1A). The periplasmic gate structure found in VcGspD, which bisects the chamber and effectively divides it into two, is absent from PilQ (Figure 6B). A superposition of PilQ on to the 10 Å resolution cryoelectron microscopy structure of the T3SS needle complex from *Salmonella* [15] enabled a comparison with the structure of a secretin in the open form. The InvG secretin component from the needle complex forms a cylindrical structure which is open at both ends, to allow assembly of the needle fiber (outlined in blue in Figure 6C). Such a comparison suggests that both the top and bottom parts of PilQ must open up to allow passage of the type IV pilus fiber, in keeping with our previous observation that TFP can bind into the PilQ chamber when added *in vitro* [43]. Direct comparisons of domain assignments to respective density maps were complicated by possible differences in detergent mass associated with the transmembrane regions, and the large amount of predicted coil or unstructured polypeptide in secretin sequences, with associated uncertainty about the degree to which these regions may contribute to observed density. Nevertheless, it is clear that significant structural differences exist between different secretin types, and also that such structures must be dynamic to allow passage of secreted pilus fibers and exoprotein substrates.

### Docking of PilQ domain structures into the cryoelectron microscopy density map and assembly of the PilQ:PilP dodecameric complex

**Figure 7:**
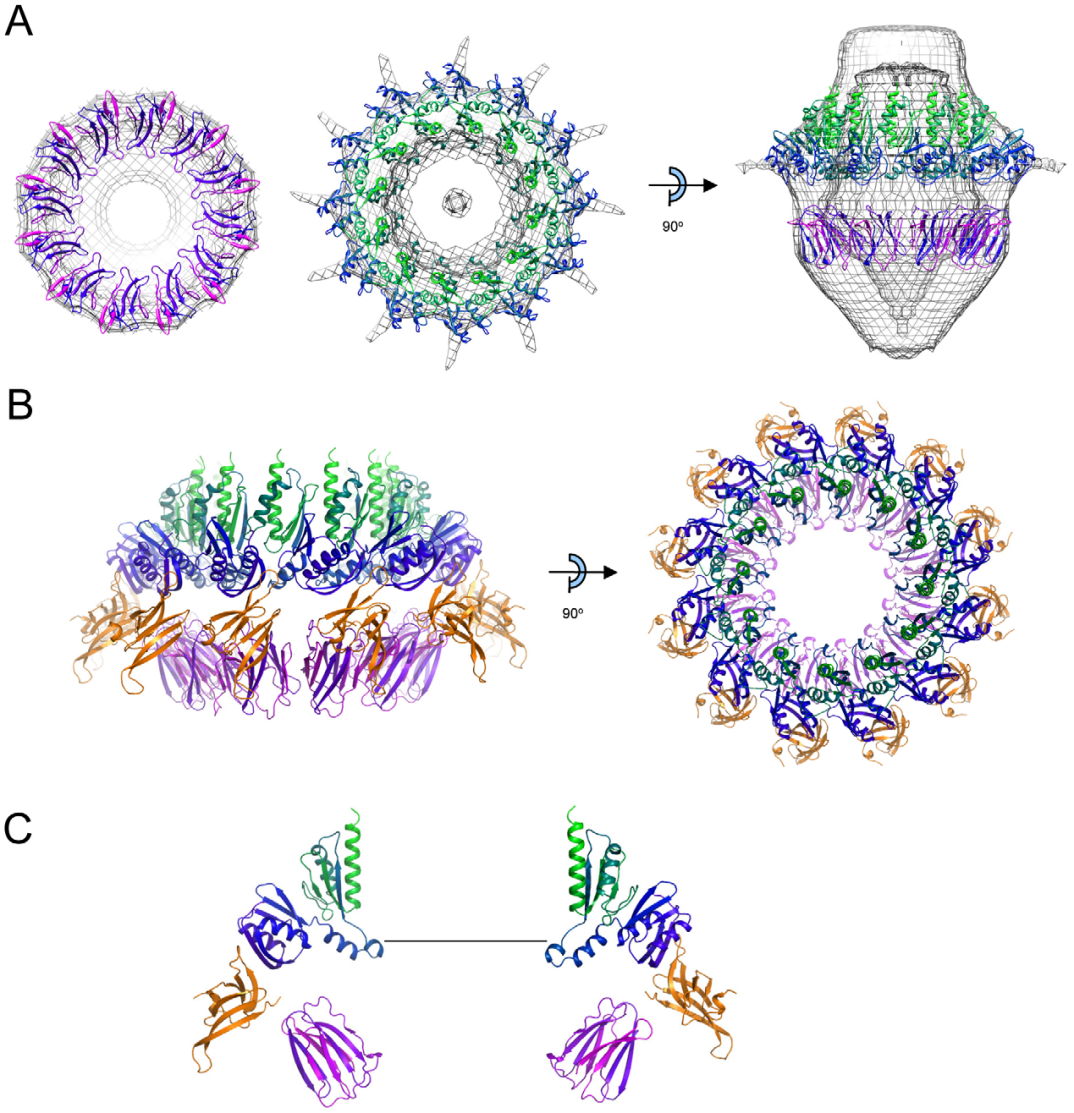
Model for the PilP-PilQ assembly. A) Docking of B2 domain (left panel), N0/N1 domain (middle panel) and both (right panel) into the PilQ cryoelectron density map, contoured at 2.9σ. Some parts of the density map and oligomers have been removed for clarity. Colors are as used in Figure 2A (B2 domain) and Figure 3B (N0/N1 domains). B) Reconstruction of PilQ N0/N1/B2 domain structures (colored as in A) with PilP C-domain (orange) bound. Left panel: side view with 6 oligomers; right panel: top view with 12 oligomers. C) Detail of two oligomers on opposing sides of the PilQ chamber. The scale bar is 60 Å and corresponds to the approximate dimensions of an assembled type IV pilus fiber.

Structures of the B2 domain (B2PilQ^224–329^) and N0/N1 double domain (N0N1PilQ^343–545^) were docked into the cryoelectron density map using MULTIFIT, a program which has been shown to work well for structures with multiple components, even with low resolution maps [44]. In addition to optimal fit to the density and minimization of steric clashes, further constraints were applied to differentiate between multiple potential solutions. First, fitting was confined to the relevant sections of the map for each domain, as shown in Figure 6A. Second, orientations of the N0/N1 structure which placed the N1 domain closer to the membrane-spanning region were favoured. Third, the PilP binding site needed to be exposed on the outer surface, in keeping with our previous demonstration that this is the case [27]. Some orientations were also precluded because they created steric clashes between PilP and adjacent PilQ molecules. Fourth, the distance between the C-terminus of the second β-domain and the N-terminus of the N0/N1 double domain needed to be lower than the maximum span which could be plausibly bridged by the missing residues. This latter criterion ruled out an ‘inverse’ orientation of the second β-domain, in which the direction of the last β-strand is towards the base of the PilQ oligomer (i.e. the N-terminal end). These constraints were applied to the highest scoring solutions obtained from MULTIFIT [44], and succeeded in identifying a unique solution for the locations of both B2PilQ^224–329^ and N0N1PilQ^343–545^ which satisfied all the criteria (Figure 7A). A striking feature of the resulting assembly is the location of the C-terminal helix in the N1 domain, which is orientated vertically, lining the sides of the top of the chamber and presumably forming a link to the transmembrane domain at the C-terminus. Although the B2 domain fitted extremely well into the relevant part of the map, there was insufficient volume remaining to accommodate a further 12 copies of the B1 domain, if it is assumed that it adopts a similar folded, globular structure. As discussed above, however, our structural work on several such domains from different bacteria did not identify any that were completely folded. We therefore propose that the first β-domain adopts a partially folded structure in the PilQ oligomer, sufficient to contribute some density to the map, but have omitted it from our model as it remains poorly defined at present.

Using the model for the PilP^77–164^:N0N1PilQ^343–545^ complex determined above, the PilP C-terminal domain can be placed onto the PilQ assembly (Figure 7B). PilP projects outward from the assembled PilQ complex, in an orientation which is different from the T2SS GspD-GspC complex: in that case, GspC was placed closer towards the interior of the secretin chamber [31]. It is also readily apparent that the PilP C-terminal domain lies close to the B2 domain, essentially sandwiched as a ‘wedge’ between the N0 and B2 domains (Figure 7C). An assembled TFP fiber measures 60 Å in diameter [45]; passage of the pilus fiber would therefore require movement of the PilQ C-domain (Figure 6A), as well as the B1/B2 domains and possibly also the linker between the N0 and N1 domains (Figure 7C). One obvious function of PilP, therefore, is to stabilize the PilQ oligomer during secretion, preventing disassociation and consequent disruption of the channel.

## Discussion

Recent structural work has started to shed some light on secretins and the way in which they mediate the transition of exoproteins across the OM. A question of particular importance is how secretins are able to function in several different secretion systems. Our work here has highlighted a critical adaptation of TFP-dependent secretins which is not found in members of the family elsewhere: the presence of separate β-domains which are involved in closing the chamber at its periplasmic end. The β-domains appear to be uniquely adapted for this purpose and must, by inference from our previous observations on the filling of the PilQ chamber with TFP [43], be involved in gating the entry of pilin or an assembled pilus fiber. A prevailing theme in structural studies on secretins is the modular organisation of their domains. Here we provide evidence that, even in the central part of the chamber where the gap for passage of the pilus fiber is at its widest, there must be some movement to accommodate the pilus fiber during secretion and retraction (Figure 7C). We do note, however, that the type IV pili in *N. gonorrhoeae* can undergo a force-induced narrowing to a form with a diameter reduced by 40% [46]. We cannot exclude the possibility, therefore, that the PilQ chamber could house the pilus fiber in an intermediate and narrower state. Flexibility of movement between adjacent domains, which we have demonstrated experimentally for the N0 and N1 domains, is likely to be a critical part of secretin function. There is also evidence that secretins somehow recognize their secreted substrates [14], [47]. These observations suggest a model in which the secretins associated with different secretion systems have diversified by modification of their periplasmic domains, and it seems likely that this is where the specificity for recognition of their secreted substrates resides. Such specificity may be necessary in organisms such as *P. aeruginosa*, which have the capacity to express more than one secretin and may therefore require mechanisms to distinguish between them.

The B2 domain sequence is well conserved in PilQ sequences from other bacteria (Figure S2), suggesting that our observations can be generalised, at least to type IVa pilus-dependent secretins [2]. It is less clear, however, whether type IVb pilus-dependent secretins adopt the same domain organization as shown in Figure 1A. Sequences of BfpB, from *E. coli*, and TcpC, from *V. cholerae*, did not align well with the neisserial B2 domain sequence, leaving this as an open question at present. The type IVb pilus-dependent secretins differ in other respects: they have lipid attachment sites at the N-terminus, for example, and no readily apparent equivalent of the PilP lipoprotein.

Our previously reported structural studies on *N. meningitidis* PilQ by electron microscopy were carried out using negatively stained specimens [12], [42], whereas the current structure has been determined in the absence of stain in vitreous buffer. To date, the best structure available for the PilQ oligomer was obtained using cryo-negative stain, a procedure which involves addition of a negative stain reagent (ammonium molybdate) to the sample before freezing. The additional contrast obtained using negative staining led to a higher quoted resolution value (12 Å) than that cited here for a low contrast, unstained sample (19 Å) but the fitting of domains into a low resolution structure requires a good representation of the true distribution of protein density across 3D space. The resulting map records the molecular envelope well, but not the internal hydrophobic features of a protein which exclude the stain. The structure reported by Collins *et al.* [12] was adequate to delineate the general structural features of the PilQ oligomer but could not reliably be used for automated docking using MULTIFIT [44], or similar programs, which make no allowance for the contribution of negative stain. Additionally, positive staining of hydrophilic regions of protein may sometimes occur, resulting in an incorrect envelope and a protein deficit where protein density should actually be observed. Finally, the staining pH and ionic strength are usually under non-physiological conditions, resulting in structural changes in the protein that may be artefactual. We therefore argue that the current structure, although it is at lower resolution than that reported by Collins *et al.*, is nevertheless a much better map into which domains can be fitted. A second difference between the two structures concerns the symmetry applied: C12 symmetry was apparent in the structure studied by Collins *et al.*, but C4 symmetry was applied as a more conservative option, given that the C4 signal was stronger and the apparent partial squaring of particles within the data. Since then, much stronger evidence has emerged for C12 symmetry of secretins [14]. Application of C12 symmetry in the refinement of either C4- or C12-symmetric preliminary models led to convergence of the structure during refinement, validating the imposition of C12 symmetry on the structure presented here.

There are a number of well documented similarities between the proteins involved in TFP biogenesis and the T2SS: these include not just the secretins and cytoplasmic ATPases, but also structural components such as the cytoplasmic protein PilM, which has a similar fold to the T2SS protein EpsL [48]. Here we have shown that PilP binds to the N0 domain of PilQ in a similar manner to the recognition of the GspD secretin by GspC [31]: the analogy therefore extends from similarity in fold between the two pairs of proteins, to a similarity in their mode of recognition. This provides further weight to the view that the two secretion systems are evolutionarily related. There are also important differences between the two systems, however. GspC is a multidomain protein, with a transmembrane helix and a C-terminal PDZ domain, as well as the HR domain which is similar in fold to PilP. PilP is also membrane-associated, but through a lipid anchor which is covalently attached to its N-terminus. Between the lipid attachment site and the beginning of the globular domain fold at the C-terminus, there is a proline-rich sequence comprising some 60–70 residues which is unstructured, at least in the *N. meningitidis* protein [29]. Sequence alignments and secondary structure predictions suggest that this is also the case in other Gram-negative pathogens (not shown). Work on PilP from *P. aeruginosa* has established that it also binds to the inner membrane proteins PilN and PilO, probably through the unstructured N-terminal region [30]. This result has also been confirmed recently in *N. meningitidis* [49], and through pull-down experiments with *N. meningitidis* PilP in solution (our unpublished data). Why is it the case that expression of PilP is critical to TFP assembly in *N. meningitidis* [26]? Our structure-based model of the PilP:PilQ complex, combined with these other recent observations, suggests that it could play a key role in maintaining assembly of the PilQ oligomer during pilus fiber secretion. There would be much reduced contact between adjacent PilQ monomers in the oligomer, once the C-domain and B1/B2 domains have opened up (Figure 6A). We note that none of the secretin periplasmic domains studied to date form dodecamers when expressed separately in recombinant form, suggesting that the interactions between adjacent monomers in this part of the oligomer are generally weak. PilP, on the other hand, is linked to the inner membrane through its lipid moiety and interaction with PilO and PilN, through its flexible N-terminus [48], [49]. Our current hypothesis is that PilP is needed to maintain the integrity of the PilQ oligomer during secretion, and that it does this by effectively forming a bridge between the PilQ periplasmic domains and the inner membrane.

The large periplasmic chamber formed by PilQ is reminiscent of similar structures found in other OM protein secretory complexes. The Wza translocon for capsular polysaccharides, for example, forms a more elongated chamber but it is also sealed at the periplasmic end [50]. The type IV secretion system complex spans the entire periplasm and, in this case, is a double walled structure with an opening on the cytoplasmic side [51]. Similar studies on the TFP biogenesis system have been complicated by difficulties in isolation of correctly folded and assembled full length PilQ in recombinant form, and in reconstituting the core secretion platform from purified inner and OM components. Our deconstruction of the PilQ-PilP binding site and ability to reassemble the PilQ-PilP complex therefore represents a first, but crucially important, step on the pathway to reassembling this complex molecular machine.

## Materials and Methods

### Protein expression and purification

Protocols for expression and purification of all proteins used in this study are described in Text S1.

### NMR Spectroscopy

#### Data collection

PilP and PilQ samples prepared for NMR analysis consisted of natural isotopic abundance or 98% ^15^N and 99% ^13^C labelled protein (1 mM-250 µM) in 500 µl of 90% ^1^H_2_O 10% ^2^H_2_O (or 100% ^2^H_2_O for specific experiments on N0N1PilQ^343–545^) in a solution containing 50 mM NaCl and 50 mM sodium phosphate at pH 6.8. All NMR experiments were carried out at 298K on Bruker Avance III 600 MHz and 800 MHz spectrometers equipped with TCI triple resonance cryoprobes. Spectra were processed using Topspin2.1 (Bruker) and the Azara processing package provided as part of the CCPN suite with assignment carried out using CCPN Analysis [52]. Triple resonance assignment was obtained utilising two-dimensional ^1^H^13^C and ^1^H^15^N HSQCs in conjunction 3D HNCA, HN(CA)CB, HN(CO)CA, HNCO, CBCA(CO)NH, HBHANH, HBHA(CO)NH, CB(CGCD)HD and HCCH-TOCSY experiments. Distance restraints were obtained from 3-dimensional ^1^H ^1^H ^13^C NOESY and ^1^H ^1^H ^15^N NOESYs using a mixing time of 100 ms. In addition, a set of experiments were collected on N0N1PilQ^343–545^ in 100% ^2^H_2_O; ^1^H^13^C HSQC, ^1^H^15^N HSQC, HCCH-TOCSY and ^1^H ^1^H ^13^C NOESY. Chemical shifts were referenced to DSS (4,4-dimethyl-4-silapentane-1-sulfonic acid). Assigned chemical shifts of the first β-domain (B1) from *Aeromonas hydrophilia*, B2PilQ^224–329^, N0PilQ^343–442^ and N0N1PilQ^343–545^ were deposited in the BioMagResBank and assigned accession numbers 18419, 18459 and 18428 respectively. The Chemical Shift Index [53] of each protein was used to determine degree of secondary structure.

#### NMR solution structure calculation & validation

Automated NOESY assignment and preliminary structure calculations of B2PilQ^224–329^ and N0PilQ^343–442^ were performed using CYANA 2.1 software [54], [55], with input data of shift lists derived from ^15^N- and ^13^C-HSQC spectra. For B2PilQ^224–329^ a total of 4260 unassigned NOESY peaks were picked of which 3881 were assigned. For N0PilQ^343–442^ a total of 2677 unassigned NOESY peaks were picked of which 2337 were assigned. CYANA 2.1 calculations were ran with standard protocols using 7 cycles of automated NOE assignment and structural calculations, producing 100 structures per cycle. Structures were subsequently water-refined using CNS1.2 [56] with a total of 2607 unambiguous interproton distance restraints and 154 dihedral restraints for B2PilQ^224–329^ and a total of 1309 unambiguous interproton distance restraints and 110 dihedral restraints for N0PilQ^343–442^. Dihedral φ and ψ torsion angles were produced by TALOS+ [57] and the final ensembles of the best 20 water-refined structures were selected on the basis of low total and NOE energies, and validated with PROCHECK-NMR [58] using the iCing interface (http://nmr.cmbi.ru.nl/icing/iCing.html). Atomic coordinates and NMR restraints of B2PilQ^224–329^ and N0PilQ^343–442^ have been deposited in the Protein Data Bank under the accession codes 4AQZ and 4AR0 respectively. The secondary structure of B2PilQ^224–329^ was calculated using the STRIDE webserver [59], [60]. Surface analysis employed NACCESS (Hubbard, S.J. & Thornton, J.M. (1993), ‘NACCESS’, Computer Program, Department of Biochemistry and Molecular Biology, University College London) for identification of exposed hydrophobic residues. CCP4MG [61] was used for calculation and display of electrostatic surface potentials, and Pymol (The PyMOL Molecular Graphics System, Version 1.3, Schrödinger, LLC) for secondary structure and side chain analysis. Schematic representations of the secondary structure layout were built using TOPDRAW [62]. Characterization of the domain fold was carried out using SCOP [33]; alignment of the CS-domain family employed PROMALS3D [63] for secondary structure-driven sequence alignment and MULTIPROT [64] for homologous structure alignment. Random coil index (RCI) analysis was carried out using the RCI webserver [37].

#### N0N1PilQ^343–545^ model

The structure of the N1 domain (N1PilQ^419–514^) could not be determined using conventional methods due to the low number of unambiguous NOEs. Therefore 'C, H_a_, C_a_, C_b_, N, and H_N_ assigned chemical shifts were submitted to the CS-ROSETTA webserver [65] available on the eNMR grid. The C terminus domain boundary of N1PilQ^419–514^ was identified using RCI [37]. To check the suitability and reliability of the CS-ROSETTA approach, the structure of N0PilQ^343–442^ was also determined using CS-ROSETTA and compared with the NOE-derived structure. The helical linker region was included for both the N0PilQ^343–442^ and N1PilQ^419–514^ ROSETTA structure determination; in both cases the linker region consisted of a four turn helix comprising P419-E432.

A complete model of the N0N1PilQ^343–545^ double domain was assembled using MODELLER [66] in multiple template mode. For the N0 domain the NMR structure was used as a template and for the linker region the ROSETTA model structures spanning PilQ residues 419–437 were used. For the N1 domain, homologous domains from the EscC and GspD structures (3GR5 residues 105–173 and 3EZJ residues 102–168 respectively), together with the ROSETTA model, were used. To eliminate orientation bias between both the N0 and N1 domains and the linker, each template consisted of only one folded domain. As no restrictions were placed on interdomain orientation, 100 structures were calculated and clustered accordingly. These 100 structures were divided into 7 clusters based on the criterion that the RMSD of the cluster be no more than 3 Å and each cluster must comprise four or more structures. For each cluster a representative model was selected as the closest to mean structure. The best approximate orientation was then selected from these structures based on quality of fit to the electron microscopy density map using MULTIFIT [44], as described in the main text.

#### Titrations

For the PilP- PilQ titration, a low concentration (50 µM) sample of ^15^N-labelled PilP^77–164^ was prepared in 50 mM NaCl and 50 mM sodium phosphate at pH 6.8. The titration was carried out to yield the following ratios of [PilP]:[PilQ]; 1∶0.1, 1∶0.2, 1∶0.5, 1∶0.8 and 1∶1 with a final concentration of 40 µM PilP^77–164^. 2D ^1^H ^15^N HSQC experiments were carried out with 2 scans, 256 increments. To ensure peaks were not lost due to dilution effects at higher titration points two datasets were acquired, one with low and the other with higher numbers of scans; in all cases, it was possible to confirm that the loss of peaks was due to binding rather than dilution. The reverse experiments using natural isotopic abundance PilP^77–164^ and ^15^N ^13^C PilQ (N0PilQ^343–442^ or N0N1PilQ^343–545^) again identified a set of peaks that attenuated upon binding. Spectra were collected at titration points of [PilQ]:[PilP]; 1∶0.1, 1∶0.2, 1∶0.3, 1∶0.5 and 1∶1. All spectra were collected and processed using TOPSPIN 2.1 (Bruker, Biospin).

#### PilP^77–164^ assignment and binding site mapping

PilP^77–164^ ^1^H ^15^N HSQC was assigned by transfer from BMRB star file 7209 [38] using CCPN format converter and CCPN analysis [52]. 93% NH backbone assignment was achieved for residues 79–163 based on closest singly matched peaks between BMRB reference assignment and experimental data. Peaks affected by the binding of PilQ domains were mapped onto the PilP structure and color-coded according to the concentration ratio where the backbone NH peak was attenuated.

#### Generation of a structural model for the N0N1PilQ^343–545^: PilP^77–164^ complex

Initial protein-protein docking utilized the restraints-driven docking program HADDOCK (*H*igh *A*mbiguity *D*riven biomolecular *DOC*King) [39], [40] with the NMR structures of N0PilQ^343–442^ and the C-domain of PilP (PDB 2IVW) [29]. AIR restraints generated from peak attenuation measured during titration were used as constraints for the rigid body docking. Five clusters were produced (with a 7.5 Å cutoff), one of which, closely resembled the equivalent GspC-GspD complex (PDB 3OSS.pdb). This latter structure was used to guide the identification of five intermolecular hydrogen bond restraints in the PilQ-PilP complex; ^PilP^100_N_-_O_351^PilQ^, ^PilP^102_N_-_O_349^PilQ^, ^PilP^102_O_-_N_349^PilQ^, ^PilP^ 100_O_-_N_351^PilQ^, ^PilP^98_O_-_N_353^PilQ^. Using CNS1.2 [56], 100 structures were then calculated using these additional restraints, together with the intramolecular NOE restraints obtained from the N0PilQ^343–442^ and PilP (PDB 2IVW) structures. An ensemble comprising the 20 lowest energy structures was obtained. The advantage of this dual HADDOCK-CNS approach was that it overcame problems associated with rigid-body docking.

### Cryoelectron microscopy and image analysis

3 µl samples were applied undiluted to Quantifoil R 1.3/2 holey carbon-coated EM grids and blotted using Whatman No.1 filter paper (2×1 sec blots) at 90% humidity and then frozen in liquid ethane using a Vitrobot plunge freezing system (FEI, Hillsboro, OR). Cryo-EM was performed using a Tecnai F20 200 kV EM operating in low dose mode at 200 kV. Micrographs were recorded using a Gatan 4 k×4 k CCD at underfocus in the range 1–5 µm and with a calibrated magnification corresponding to 4.53 Å/pixel at the specimen level. Images were recorded under low-dose mode with an overall electron dose of 20- 25 electrons/Å^2^. Particles were selected into 64×64 pixel boxes (equivalent to 290×290 Å) from the digital micrographs using the EMAN software package [67] and masked with a circular mask of radius 131 Å. After correction of the microscope contrast transfer function (CTF), and removal of outlier particles (based on size), a final dataset of 25,303 particles were used to calculate the low resolution 3D structure of PilQ. An initial model was generated by selection of small (<0.5%) subsets of particles with the strongest n-fold symmetry and strongest bilateral symmetry, and then calculating a noisy 3D structure assuming an orthogonal relationship between the two sets of particles. (EMAN command *startcsymm*). Based on prior work [12] we generated preliminary models for both C4 and C12 symmetry. Iterative refinement of the initial structures was subsequently carried out using the entire dataset, and using both C4 and C12 symmetry for refinement of each model. Comparison of projections of the 3D structures with the corresponding particle class averages, showed a good agreement with the C12 symmetric structure (Figure 5). Moreover applying C12 symmetry in the refinement of either C4- or C12-symmetric preliminary models led to convergence. Estimation of the resolution of the final structure using the same method applied by Collins *et al.* [12], measuring the value at which a comparison of the Fourier shell correlation (FSC) of one half of the dataset with the other reaches 0.5, gave a value of 1/19 Å^−1^. Application of the more recently introduced, and more conservative, *rmeasure* software [68], gave a value of 1/33 Å^−1^ resolution. Maps derived by electron microscopy were displayed with the CHIMERA software package [69]. The PilQ density map was deposited in the EMDataBank with accession code EMD-2105 and coordinates for the modelled PilQ:PilP complex are available as PDB deposition 4AV2.

## Supporting Information

Figure S1 **Comparison of the folds of the B2 domain from** ***N. meningitidis*** **PilQ (B2PilQ^224–329^) with the CS domain from human Sgt1.** The second β-domain is shown on the left, in green, and the CS domain on the right, in light blue (PDB accession code 1RL1).
(TIF)

Figure S2 **Structure-based sequence alignment of B2 domains.** The locations of β-strands in the *N. meningitidis* structure are shown. Numbering is for the *N. meningitidis* sequence. Residues which are well conserved are highlighted in bold. Example sequences shown are *Pseudomonas aeruginosa* (Pa), *Xanthomonas campestris* (Xc), *Aeromonas hydrophila* (Ah), *Legionella pneumophila* (Lp) and *Xylella fastidiosa* (Xf) (Uniprot codes A3L2L4, B0RPC1, A0KN30, Q6VY32 and B2I8B2 respectively).
(TIF)

Figure S3 **Chemical shift and deduced secondary structure assignments for the B1 domain from** ***Aeromonas hydrophila*** **.** Top: CSI calculated for deviations from random coil shifts of Hα, Cα and CO to determine the consensus secondary structure, graph adapted from CCPN analysis. Bottom: alignment of B1 domain in *A. hydrophila* (Ah), *N. meningitidis* (Nm), *P. aeruginosa* (Pa) *and L. pneumophila* (Lp) (Uniprot codes A0KN30, Q70M91, A3L2L4, and Q6VY32 respectively). Residue sequence and numbering for the *A. hydrophilia* sequence incorporates the loss of the signal sequence and residues from the expression vector.
(TIF)

Figure S4 **Chemical shift and deduced secondary structure assignments for N0N1PilQ^343–545^ and N0PilQ^343–442^ from** ***Neisseria meningitidis*** **.** CSI calculated for deviations from random coil shifts of Hα, Cα and CO to determine the consensus secondary structure; graph adapted from CCPN analysis.
(TIF)

Figure S5 **Ratio of PilQ:PilP at the point of PilQ NH peak attenuation in the PilQ N0 and N1 domains on binding of PilP^77–164^.** Binding of PilP^77–164^ to N0N1PilQ^343–545^ is shown in blue and N0PilQ^343–442^ in red.
(TIF)

Text S1 **Protein expression and purification.** Methods for the expression and purification of all proteins used in this study.
(PDF)
