# Supplementary figures and images for: Structure and Assembly of a Trans-Periplasmic Channel for Type IV Pili in Neisseria meningitidis
Source: PLoS Pathog. 2012 Sep 13;8(9):e1002923. doi: 10.1371/journal.ppat.1002923 (PMC3441751; doi:10.1371/journal.ppat.1002923)

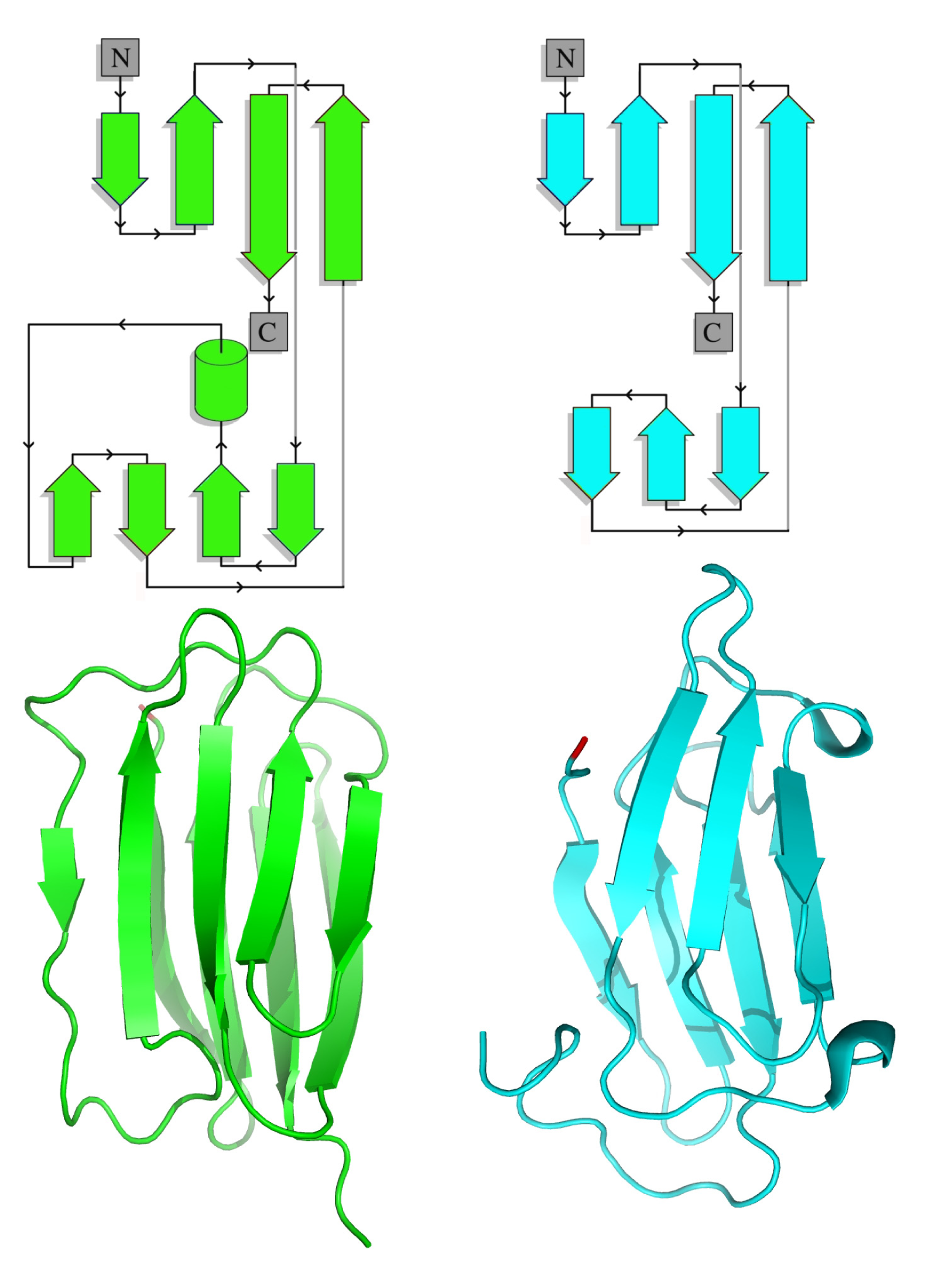

Supplement: Figure S1 — Comparison of the folds of the B2 domain from N. meningitidis PilQ (B2PilQ224–329) with the CS domain from human Sgt1. The second β-domain is shown on the left, in green, and the CS domain on the right, in light blue (PDB accession code 1RL1). (TIF) [file ppat.1002923.s001.tif]

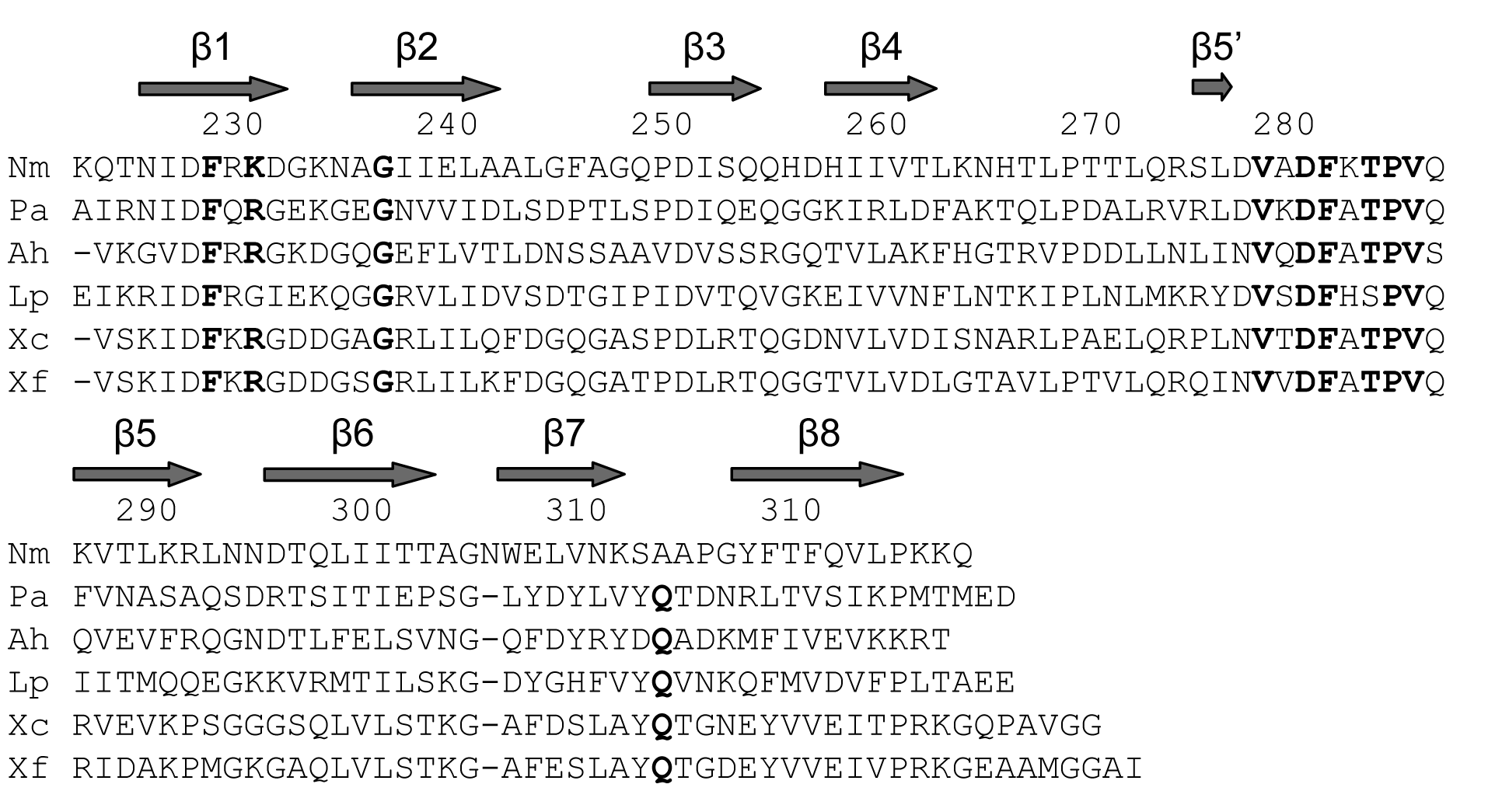

Supplement: Figure S2 — Structure-based sequence alignment of B2 domains. The locations of β-strands in the N. meningitidis structure are shown. Numbering is for the N. meningitidis sequence. Residues which are well conserved are highlighted in bold. Example sequences shown are Pseudomonas aeruginosa (Pa), Xanthomonas campestris (Xc), Aeromonas hydrophila (Ah), Legionella pneumophila (Lp) and Xylella fastidiosa (Xf) (Uniprot codes A3L2L4, B0RPC1, A0KN30, Q6VY32 and B2I8B2 respectively). (TIF) [file ppat.1002923.s002.tif]

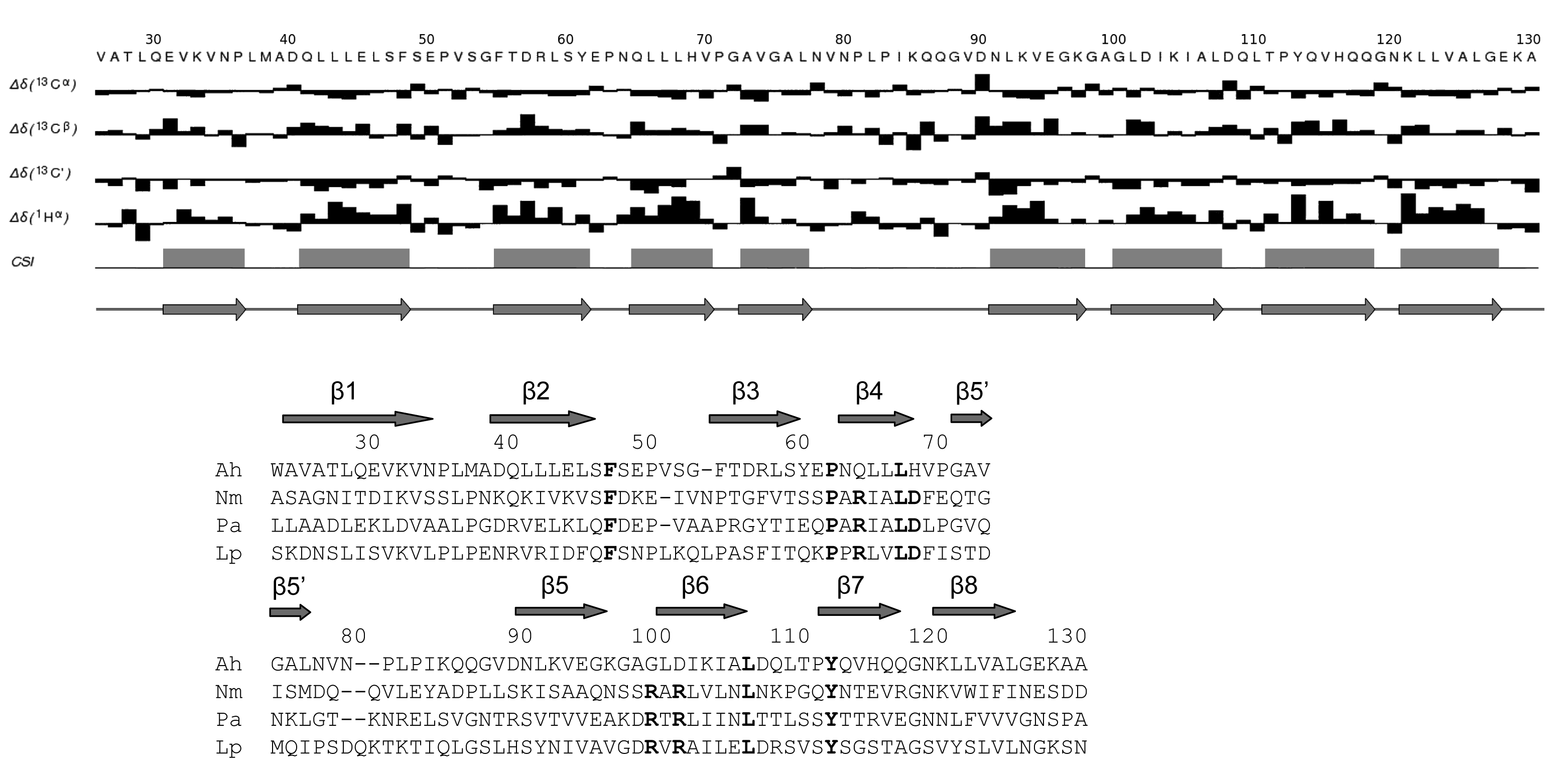

Supplement: Figure S3 — Chemical shift and deduced secondary structure assignments for the B1 domain from Aeromonas hydrophila . Top: CSI calculated for deviations from random coil shifts of Hα, Cα and CO to determine the consensus secondary structure, graph adapted from CCPN analysis. Bottom: alignment of B1 domain in A. hydrophila (Ah), N. meningitidis (Nm), P. aeruginosa (Pa) and L. pneumophila (Lp) (Uniprot codes A0KN30, Q70M91, A3L2L4, and Q6VY32 respectively). Residue sequence and numbering for the A. hydrophilia sequence incorporates the loss of the signal sequence and residues from the expression vector. (TIF) [file ppat.1002923.s003.tif]

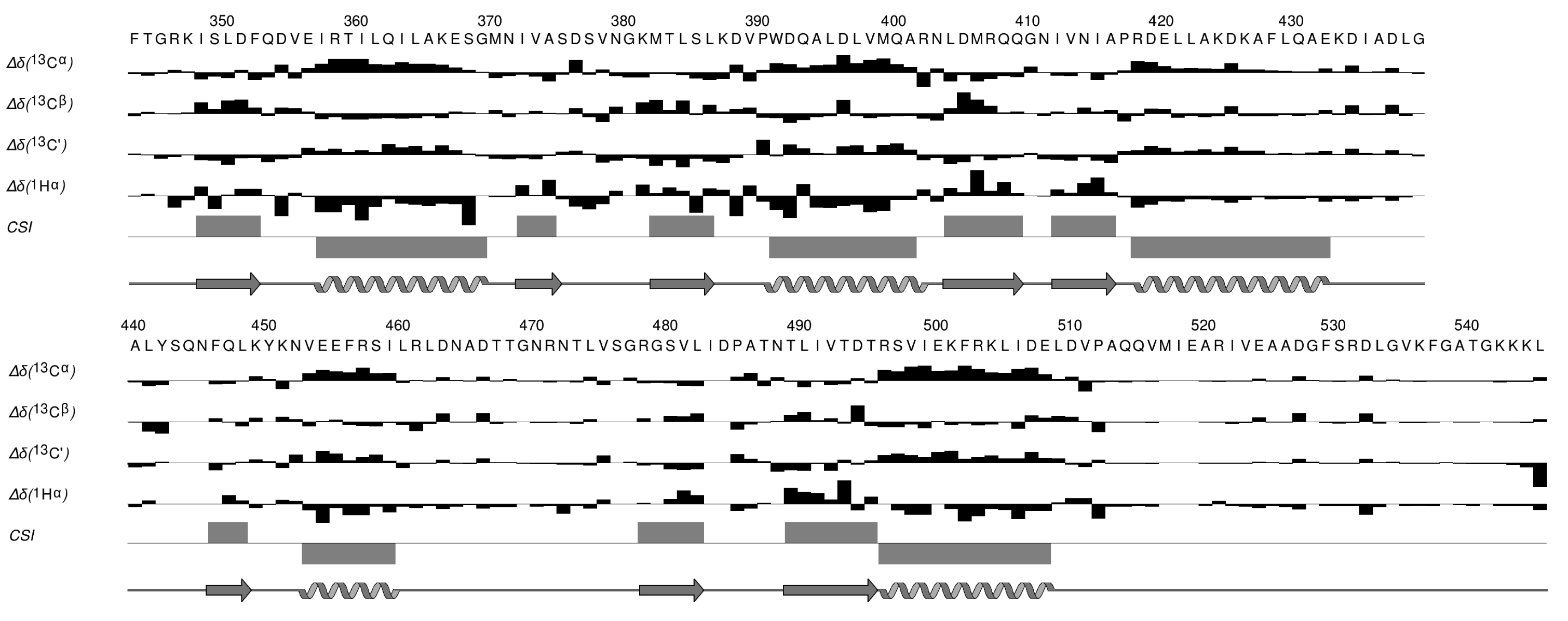

Supplement: Figure S4 — Chemical shift and deduced secondary structure assignments for N0N1PilQ343–545 and N0PilQ343–442 from Neisseria meningitidis . CSI calculated for deviations from random coil shifts of Hα, Cα and CO to determine the consensus secondary structure; graph adapted from CCPN analysis. (TIF) [file ppat.1002923.s004.tif]

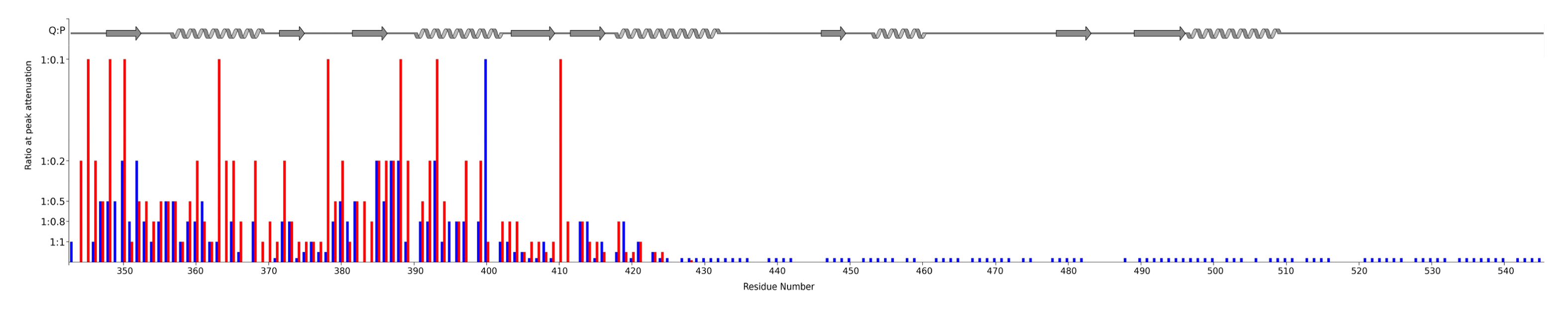

Supplement: Figure S5 — Ratio of PilQ:PilP at the point of PilQ NH peak attenuation in the PilQ N0 and N1 domains on binding of PilP77–164. Binding of PilP77–164 to N0N1PilQ343–545 is shown in blue and N0PilQ343–442 in red. (TIF) [file ppat.1002923.s005.tif]
