# Supplementary material for: Structure and Assembly of a Trans-Periplasmic Channel for Type IV Pili in Neisseria meningitidis
Source: PLoS Pathog. 2012 Sep 13;8(9):e1002923. doi: 10.1371/journal.ppat.1002923 (PMC3441751; doi:10.1371/journal.ppat.1002923)
Supplement: Text S1 — Protein expression and purification. Methods for the expression and purification of all proteins used in this study. (PDF) [file ppat.1002923.s006.pdf]

Protein expression and purification

a) PilP$^{77-164}$

The DNA sequence encoding *Neisseria meningitidis* C-terminal PilP was amplified from strain MC58 genomic DNA using the primers CCGACAAAAAACATATGAATGCCCCCGACACCAAG and GTTCGGTGTTTTTGGATCCTTAATTCAACAGCAGTTCTG. The PCR product was digested using *NdeI* and *BamHI* and ligated into the pET15b expression vector (Novagen), to generate a construct coding for a polypeptide with a hexahistidine tag at the N-terminus and a thrombin cleavage site. The resulting construct was designated pET15b_PilP$^{77-164}$. For protein expression, pET15b_PilP$^{77-164}$ was transformed into *E. coli* T7 Express cells (NEB) and grown in 2x-YT medium plus 75μg ml^{-1} ampicillin; a 50ml starter culture was grown at 37°C for 4 hours and then used to inoculate 450ml of 2x-YT plus 75μg ml^{-1} ampicillin which was grown, with shaking, at 37°C until the absorbance of the media at 600nm reached 0.6. Isopropyl-D-thiogalactoside (IPTG) was added to a final concentration of 0.1mM and the cells were grown for 18 hours at 20°C. For the preparation of ^{15}N-labelled material, the 50ml 2x-YT starter culture was sedimented by centrifugation at 6,000g_{av} for 20 mins at 4°C, washed once with sterile water, and then used to inoculate 450ml M9 minimal medium, prepared using ^{15}N-NH_4Cl and supplemented with 100mg ^{15}N-Isogro (Sigma). The cultures were grown to mid-log phase before induction with 1mM IPTG for 16 hours at 37°C. Typically, four 500ml cultures were used per protein purification run. Following growth, cells were harvested by centrifugation at 6,000g_{av} for 20 mins at 4°C and each pellet (from 500ml media) was re-suspended in 10ml of lysis buffer (50mM NaH_2PO_4/Na_2HPO_4 pH 7.8, 100 mM NaCl) containing 1x EDTA free complete proteinase inhibitor (Roche) and 1mg ml^{-1} lysozyme (Sigma). Cells were disrupted by sonication at 4°C, and insoluble debris sedimented by centrifugation for 20 minutes at 12,800g_{av} at 4°C. The supernatant was collected, treated with 0.04 mg ml^{-1} DNase and passed through a 0.45μm filter, before a second centrifugation for 30 minutes at 18,700g_{av} at 4°C. 500μl Ni-IDE metal chelate resin (Generon), which had been pre-equilibrated with lysis buffer, was added to the soluble fraction along with imidazole to a final concentration of 10mM. The suspension was incubated with constant gentle agitation for 2 hours at 4°C, before being applied to a gravity flow disposable column (BD Biosciences). After collection of unretained eluent, the resin was washed with 1.5ml wash buffer (lysis buffer plus 20mM imidazole) and bound protein eluted in 1ml elution buffer (lysis buffer plus 200mM imidazole). Imidazole was then removed by dialysis at 4°C against 2L of lysis buffer for 8 hours, and the buffer was replaced once halfway through this period. The purification tag was not removed from PilP$^{77-164}$; therefore the polypeptide comprised 109 residues, beginning with the intact hexahistidine tag and thrombin cleavage site MGSSHHHHHHSSGLVPRGSHM, followed by the PilP$^{77-164}$ polypeptide beginning with APDTK…. and ending with ...ELLLN. The protein was dialysed against 50mM NaH_2PO_4/Na_2HPO_4 (pH 6.8), 100mM NaCl for NMR studies.

b) *Neisseria meningitidis* PilQ domains: N0N1PilQ$^{343-545}$, N0PilQ$^{343-442}$, B2PilQ$^{224-329}$ and B1B2PilQ$^{24-329}$

The DNA coding regions of the PilQ gene for individual domains used in this study was amplified from *Neisseria meningitidis* (strain MC58) genomic DNA using the following primers:

N0N1PilQ$^{343-545}$: GAACAATGCGCCCGGATCCTTCACAGGCCGGAAAATC and GAATGCGCTTGTCTCGAGTTACAGCTTTTTCTTGCC.

N0PilQ$^{343-442}$: GGCGCCATGGGGTTCACAGGCCGGAAAA and CGGATCCGGTACCTTAATACAGCGCACCCAAA.
B2PilQ$^{224-329}$: CGCCTTTCAGACAGGATCCGCAGGAAACATTACA and CACGCCGCCTGACTCGAGTTATTGTTTTTCGGCAG
B1B2PilQ$^{24-329}$: CAGGCGGCAGCAGGATCCAAACAAACCAATATC and CACGCCGCCTGACTCGAGTTATTGTTTTTCGGCAG

PCR products of N0N1PilQ$^{343-545}$, B2PilQ$^{224-329}$ and B1B2PilQ$^{24-329}$ were digested with *BamHI* and *XhoI* restriction endonucleases, and ligated into the pET28a expression vector to incorporate an N-terminal hexahistidine purification tag. PCR products of N0PilQ$^{343-442}$ were digested with *NcoI* and *KpnI* restriction endonucleases, and ligated into the pET11 expression vector to incorporate an N-terminal hexahistidine purification tag. The expression protocol in each case was identical for that used for PilP$^{77-164}$, except that each 500ml culture was induced with 0.5mM IPTG for 3 hours at 37°C, in the presence of 40μg ml^{-1} kanamycin. The purification procedure was the same as that for PilP$^{77-164}$, and the proteins were also dialysed against 50mM NaH_2PO_4/Na_2HPO_4 (pH 6.8), 100mM NaCl after elution from the nickel column. The purification tags were not cleaved.

c) The first β-domain from *Aeromonas hydrophila* (AhPilQ$^{26-130}$)
AhPilQ$^{26-130}$ was one of a group of constructs which were cloned and tested for protein expression using the specialized high-throughput approaches at the Oxford Protein Production Facility (OPPF) [1]. In summary, PCR primers were first designed for each construct for In-Fusion cloning into the pOPINF expression vector. The vector introduces an N-terminal histidine purification tag and a 3C-protease cleavage site. Initial PCR reactions for all constructs were set up in 96-well plates using the KOD Xtreme Hot Start DNA Polymerase master mix (Novagen) and these were analysed by AGE. A second round of PCR was then conducted for problematic targets with altered parameters using Phusion Flash master mix (NEB). PCR products were then purified by AMPure XP magnetic bead-based purification. Ligation reactions were set up simultaneously in 96-well plates, by transferring 10-250ng of the purified PCR product to a dry-down In-Fusion plate of lyophilized enzyme and buffer and incubation for 30 minutes at 42°C. Ligation products were transformed into OmniMaxII cloning grade *E. coli* and verified by blue-white screening, mini-plasmid preparation using a QIAGEN BioRobot 8000, and PCR. This resulted in positive clones for the full 47 target sequences. All 47 constructs plus a control expressing GFP were transformed into both *E. coli* B834 and *E. coli* Rosetta pLacI for small scale expression trials. For IPTG induction, single colonies were grown in 0.5 ml starter cultures in four 24-well deep-well blocks overnight. These were used to inoculate 2.5ml cultures which were grown to OD^{600} of ~0.5 before induction. These were induced with 0.5mM IPTG overnight in an orbital shaker at 20°C. All constructs were also tested for expression in both strains using Overnight Express Instant TB (TB-ONEX) auto-induction medium (manufacturer), in the same manner but without addition of IPTG. These were induced for 20-24 hours at 25°C. Cells were harvested by centrifugation within the block plates, and pellets were broken by freeze-thawing and resuspension in lysis buffer containing lysozyme and DNase I. The soluble fractions were transferred to micro-titre plates and the His-tagged protein of interest was purified by IMAC robotically using a QIAGEN BioRobot 8000 instrument according to the OPPF standard protocol.

d) Isolation of native *Neisseria meningitidis* PilQ oligomer
PilQ complex was purified from outer membranes of *N. meningitidis* serogroup B strain M1080 by detergent solubilization and size exclusion as previously described by Collins et al.[2].

1. Berrow NS, Alderton D, Sainsbury S, Nettleship J, Assenberg R, et al. (2007) A versatile ligation-independent cloning method suitable for high-throughput expression screening applications. Nucleic Acids Res 35: e45.
2. Collins RF, Frye SA, Kitmitto A, Ford RC, Tonjum T, et al. (2004) Structure of the *Neisseria meningitidis* outer membrane PilQ secretin complex at 12 Ångstrom resolution. J Biol Chem 279: 39750-39756.
